# *Streptococcus agalactiae* in childbearing age immigrant women in Comunitat Valenciana (Spain)

**DOI:** 10.1038/s41598-020-66811-2

**Published:** 2020-06-18

**Authors:** José Miguel Sahuquillo-Arce, Alicia Hernández-Cabezas, María Jesús Castaño-Aroca, Rabab Chouman-Arcas, Estefanía Díaz-Aguirre, Beatriz Acosta-Boga, José Luis López-Hontangas

**Affiliations:** 1 Microbiology Department, Hospital Politécnico y Universitario La Fe, Valencia, Spain; 2 Respiratory Infections Research Group, IIS La Fe, Valencia, Spain; 3 Universidad Miguel Hernández, Elche, Spain

**Keywords:** Clinical microbiology, Risk factors

## Abstract

*Streptococcus agalactiae* (GBS) remains the leading cause of meningitis and neonatal sepsis in the world, and causes disease in pregnant and puerperal women. This is a retrospective study of GBS infections on women of childbearing age living in Comunitat Valenciana, Spain (years 2009–2014) and GBS colonization rate on pregnant women attending Hospital La Fe (years 2013–2015) according to their origin. An aggregated total of 6,641,960 women exposed during the study period had an average GBS isolation rate of 5.19‰ (5.14–5.25‰), geographical group rates being: Western Europe (2.2‰), North America (2.1‰), Australia (3.7‰), Spain (4.6‰), Latin America II (4.5‰), Eastern Europe (5.3‰), Asia (6.7‰), Latin America I (7.7‰), Middle East (7.9‰), Indian Subcontinent (17.2‰), North Africa (17.8‰), Sub-Saharan Africa (22.7‰). The 4532 pregnant women studied had an average GBS colonization rate of 12.47% (11.51–13.43) and geographical group rates varied similar to geographical isolation rates. Low GDP and high temperatures of the birth country were associated with higher colonization rates. Thus, differences in GBS colonization depend on the country of origin; Africa and the Indian subcontinent presented the highest, while Western Europe and North America had the lowest. This variability portrays a geographical pattern influenced by temperature and GDP.

## Introduction

*Streptococcus agalactiae*, also known as Group B *Streptococcus* (GBS), is a commensal of the gastrointestinal tract and vagina of a high proportion of healthy adults. GBS remains the leading cause of meningitis and neonatal sepsis in the world, affecting 0.5 to 3 newborns in every 1000 live births. But GBS also causes disease in pregnant and puerperal women such as chorioamnionitis, preterm birth or even stillbirth^1,2^. The newborn is colonized by GBS as it passes through the birth canal, which occurs in approximately 40–60% of the children of carrier mothers, and 1–2% of them develop an infectious condition with high morbidity and mortality rates^3–5^. In addition, neonatal colonization rates are directly proportional to the mother’s vaginal colonization density and inversely proportional to the titer of antibodies against the colonizing strain^6^.

The screening of GBS in pregnant women is fundamental to knowing the state of vaginorectal colonization and establishing intrapartum antibiotic prophylaxis to reduce the risk of developing neonatal invasive infection^7–9^. The Pregnancy Monitoring Program in Comunitat Valenciana incorporated the screening of GBS colonization in 2002 according to a consensus review of a previous circular (1/97 of April 17). This was reflected in the manual for healthcare professionals entitled “Basic Pregnancy Control in Comunitat Valenciana”, based on international recommendations. Therefore, since 2002, screening of GBS in the Comunitat Valenciana is carried out on pregnant women between weeks 35 and 37.

After the widespread implementation of GBS screening and the administration of intrapartum antibiotic prophylaxis, the incidence of early onset neonatal invasive infection due to GBS has decreased more than 80% in Europe and the USA^1,10^. However, the incidence of late onset neonatal invasive infection has remained stable about 0.25–0.5 per 1000 live births due to different ways of acquiring GBS^1^.

A promising alternative to these strategies which is currently undergoing multiple clinical trials is the immunization of pregnant women. Recently, published data have shown that an increase in IgG in the serum of pregnant women correlates with a decrease in the colonization of the vaginorectal area^6,11^. This would reduce the exposure of the newborn to GBS and thus the risk of early onset infection. In addition, the levels of maternal IgG in the neonates would be enough to protect them from late-onset infection^6,12^. Moreover, vaccination would also diminish GBS associated miscarriage, stillbirth and maternal infection^1^

Prior studies have demonstrated that individual GBS colonization is remarkably homogeneous and stable through time^13,14^. Therefore, the acquisition of microbiota will depend on the mother’s colonizing bacteria and the local environment where people are raised, and will probably remain the same through life unless unbalanced by direct aggressions to their structure such as antimicrobial treatments or dramatic weather changes^15–17^.

Immigrant women from all over the world reside in Comunitat Valenciana; thus, the aim of this study is to assess the prevalence of GBS colonization among pregnant women attending Hospital La Fe or associated health care centres, and to identify newborns at higher risk for GBS infection according to their mothers’ origin. For this purpose we have determined the GBS colonization rates among pregnant women from Hospital La Fe and compared it with the isolation rates of GBS in childbearing age women living in Comunitat Valenciana (Spain) to find trends or geographical variety.

## Material and Methods

This is a retrospective study to analyze the rate of GBS carriage among women of childbearing age according to their country of origin. In a first approach, pregnant women attending our hospital or associated health care centres (Health area Valencia-La Fe) during a three-year period (2013–2015) who were studied for GBS colonization were included in the study. Hospital La Fe has a total of 945 beds and 20 associated health care centres. Its health area covers a total population of 255873. In 2018, our hospital had 661423 medical appointments, 44883 medical admissions 33183 surgical procedures and 661423 medical consultations.

Vaginal and rectal samples obtained at prenatal visits or admission for delivery were cultured on selective chromogenic medium (chromID Granada agar, bioMérieux, Marcy l’Étoile, France) and incubated for 48 hours in an anaerobic atmosphere for the screening of Group B *Streptococci*. Orange colonies were further identified by Maldi-tof (Vitek MS, bioMérieux).

In a second approach, isolation rates of GBS from clinical samples in women of ages between 15 and 49 years old during a six-year period (2009–2014) and living in Comunitat Valenciana (Spain) were identified through the Microbiological Surveillance Network of the Valencian Community (RedMIVA). This network is a system that processes and records microbiological information from more than 90% of the population living in Comunitat Valenciana^18^, which had a size of 4,934,993 people as of December 2016 and about 1,020,000 childbearing age women.

Population data including age group, sex and country of origin were extracted from the Instituto Nacional de Estadística (Spanish Statistics Office). Data about co-morbidities were obtained from the Documentation department of Hospital La Fe and the Sistema de Información de la Asistencia Ambulatoria de la Conselleria de Sanitat. Explanatory co-variates tested were: age, Gross Domestic Product per capita based on purchase parity power (GDP) and the mean temperature of the country of birth. GDP and mean temperature were extracted from Weatherbase and The World Factbook^19,20^. Climate data was obtained from High-resolution gridded datasets from the University of East Anglia and GISTEMP Team, 2019: GISS Surface Temperature Analysis (GISTEMP), version 4. NASA Goddard Institute for Space Studies^21–23^.

Colonization rates in pregnant women from Hospital La Fe and isolation rates in the aggregated population were calculated with 95% confidence intervals. Subjects were grouped according to their birth country by geopolitical areas with similar prevalence. Chi square and Welch tests were used to compare rates and ages respectively. *Post hoc* analyses were used after applying Bonferroni’s correction. A p < 0.05 was considered statistically significant. Curvilinear estimation was used to construct models that fitted explanatory co-variates. SGB colonization trends during the 6-year period 2009–2014 were compared with temperature trends from the corresponding geopolitical area in thirty nine 6-year groups from 1970 through 2009. Correlations between normalised temperature trends and normalised SGB colonization trends were assessed by the Kolgomorov-Smirnov test. Data were analyzed using the SPSS software version 15.0.

The fundamental ideas behind our work are that microbiota is naturally acquired during birth and early life, that its altering depends on severe aggressions, and that populations with higher GBS isolation rates must consequently have higher GBS colonization rates.

Access to the RedMIVA databases was granted by the Public Health Department and our research was approved by the RedMVA committee. Other data acquisition and processing were carried out in accordance with relevant guidelines and regulations and under permission of the local authorities (Conselleria de Sanitat, Comunitat Valenciana) and was approved by the ethics committee of the Instituto de Investigación Sanitaria del Hospital Universitario y Politécnico La Fe. The data was fully anonymised so informed consent from participants was not required, and access was granted in line with European General Data Protection Regulations. No experiments were performed either on humans or on human tissue samples; therefore, informed consent was not needed.

## Results

### Geopolitical areas with similar prevalence

Twelve groups were formed based on similar isolation rates and geographical proximity (Table 1). The names Latin America I and II were used to group American countries with different isolation rates but in the same continent.

**Table 1:** Geopolitical grouping according to similarity among isolation rates and proximity.

GROUP	COUNTRIES	GDP^*^	Mean T(°C)
Asia	China, Indonesia, Japan, Laos, Nepal, Philippines, South Korea, Thailand, Vietnam	16078	20.7
Australia	Australia	48800	17.3
Eastern Europe	Albania, Belarus, Bosnia-Herzegovina, Bulgaria, Croatia, Czech Republic, Estonia, Hungary, Kazakhstan, Latvia, Lithuania, Moldova, North Macedonia, Poland, Romania, Russia, Serbia, Slovakia, Slovenia, Ukraine, Uzbekistan	21067	8.3
Indian Subcontinent	Bangladesh, India, Pakistan	5233	23.7
Latin America I	Bolivia, Colombia, Costa Rica, Dominican Republic, Ecuador, El Salvador, Grenade, Guatemala, Haiti, Honduras, Nicaragua, Panama, Paraguay, Peru, Venezuela	11082	23.2
Latin America II	Argentina, Brazil, Cuba, Chile, Jamaica, Mexico, Uruguay	18583	19.4
Middle East	Armenia, Azerbaijan, Georgia, Iran, Iraq, Jordan, Kuwait, Lebanon, Syria, Turkey	19110	16.1
North Africa	Algeria, Egypt, Mauritania, Morocco, Tunisia	10967	20,4
North America	Canada, United States of America	51750	7.6
Spain	Spain	36500	15.5
Sub-Saharan Africa	Angola, Benin, Burkina Faso, Cameroon, Cape Verde, Chad, Democratic Republic of the Congo, Equatorial Guinea, Ethiopia, Ghana, Guinea, Guinea-Bissau, Ivory Coast, Kenya, Lesotho, Liberia, Mali, Nigeria, Senegal, Sierra Leone, Somalia, Sudan, Tanzania, The Gambia, Togo	4392	24.9
Western Europe	Austria, Belgium, Denmark, Finland, France, Germany, Ireland, Italy, Luxembourg, Netherlands, Norway, Portugal, Sweden, Switzerland, United Kingdom	51980	8.4

^*^GDP, Gross Domestic Product per capita based on purchase parity power (US dollars.)

### Pregnant women attending Hospital La Fe

The total number of pregnant women included in the study was 4532, with an average GBS colonization rate of 12.47% (11.51–13.43%), 90.8% of the colonized women were detected after routine GBS screening between weeks 35–37, while 9.2% were detected before that period due to bacterial vaginosis or to threatened preterm labour. Ages ranged from 29.0 (±4.2) years in Asia to 34.7 (±5.7) years in Western Europe (Table 2). There were no significant age differences between colonized and non-colonized women.

**Table 2:** GBS rates and mean age in aggregated population and pregnant women from Hospital La Fe.

	Aggregated population			Pregnant women from Hospital La Fe		
Geographical group	N	IR (CI 95%)	CP Mean age (±SD)	n	CR (CI 95%)	Mean age (±SD)
North America	6060	0.0021 (0.0010–0.0033)	33.2 (±7.2)	2	0.00 (0.00–0.00)	33.0 (±4.2)
Western Europe	345399	0.0022 (0.0020–0.0024)	33.5 (±7.2)	61	0.05 (−0.01–0.10)	34.7 (±5.4)
Australia	1075	0.0037 (0.0001–0.0074)	38.0 (±3.2)	0	—	—
Spain	4940794	0.0046 (0.0046–0.0047)	32.4 (±6.7)	3179	0.11 (0.10–0.12)	33.0 (±5.2)
Latin America II	167151	0.0045 (0.0042–0.0049)	31.6 (±6.9)	79	0.09 (0.03–0.15)	33.3 (±4.9)
Eastern Europe	506452	0.0053 (0.0051–0.0055)	29.3 (±6.1)	205	0.15 (0.10–0.19)	30.3 (±5.2)
Asia	43264	0.0067 (0.0059–0.0075)	30.1 (±5.8)	63	0.10 (0.02–0.17)	29.0 (±4.2)
Latin America I	418787	0.0077 (0.0075–0.0080)	31.0 (±7.4)	550	0.15 (0.12–0.18)	30.0 (±6.6)
Middle East	15465	0.0079 (0.0065–0.0093)	29.5 (±6.1)	17	0.18 (0.00–0.36)	31.1 (±5.4)
Indian Subcontinent	12044	0.0172 (0.0149–0.0195)	29.0 (±5.0)	83	0.11 (0.04–0.18)	30.5 (±5.2)
North Africa	154073	0.0178 (0.0172–0.0185)	29.7 (±6.4)	146	0.21 (0.15–0.28)	31.7 (±5.7)
Sub–Saharan Africa	31396	0.0227 (0.0211–0.0244)	30.0 (±5.4)	147	0.31 (0.24–0.39)	31.6 (±5.1)

^*^IR, Isolation Rate; CI, Confidence Interval; CP, Culture-positive women; CR, Colonization Rate.

The prevalence of co-morbidities in our cohort of pregnant women showed the following distribution: 1(0.02%) alcohol abuse, 164(3.6%) smoking, 164(5.4%) obesity, 65 (1.4%) diabetes, 9(0.2%), 72 (1.6%) gestational diabetes, 9(0.2%), chronic liver disease, 10(0.2%) chronic obstructive pulmonary disease, 28(0.6%) chronic central nervous system disease –Multiple Sclerosis chiefly–, 30(0.7%) chronic heart diseases –heart murmurs chiefly–, 135(3%) autoimmune chronic diseases –psoriasis chiefly–, 18(0.4%) HIV positive and 2(0.04%) kidney transplants.

Significant differences were found between the colonization rates of the different groups (p < 0.0001), and *post hoc* analysis showed that Sub-Saharan Africa women, with 31% of carriers, had significantly higher rates than the other groups except North Africa, which presented a 21% of carriers. North America and Australia were excluded from the *post hoc* analysis because of the small number of cases. Pregnant women data fitted total population results in a linear regression model with an R^2^ of 0.68.

During the study period, GBS was also isolated in 2692 samples from 649(32.3%) admitted patients and 1359(67.7%) from the health area. Infections ranged from skin and soft tissue infections to more severe infections, including 2 neonatal deaths due to bacteraemia and meningitis (Table 3). All isolates tested were susceptible to beta-lactams, linezolid and vancomycin, 98% to levofloxacin, 72.9% to clindamycin and 73.4% to erythromycin according to EUCAST criteria.

**Table 3:** GBS isolation site in CBA women during the period 2009-2014 and in patients from Hospital La Fe during the period 2013-2015 (n(%)).

	2009-2014 (CBA women)	2013-2015 (All patients)
Deep specimens		
Catheter	3 (0.01)	
CSF	6 (0.01)	3 (0.11)
Biological sterile fluid	(0.04)	14 (0.52)
Bone and deep tissue	28 (0.06)	42 (1.56)
Blood	47 (0.10)	26 (0.97)
Respiratory specimens		
Lower respiratory tract	27 (0.06)	29 (1.08)
ORL	49 (0.11)	33 (1.23)
Superficial specimens		
Surgical wound	100 (0.22)	15 (0.56)
Skin and soft tissue	249 (0.55)	131 (4.87)
Urinary tract specimens		
UTI	11146 (24.59)	1013 (37.63)
Reproductive area specimens		
Mammary gland	16 (0.04)	2 (0.07)
Endometrium	25 (0.06)	2 (0.07)
Intrauterine device	31 (0.07)	2 (0.07)
Placenta and amniotic fluid	113 (0.25)	8 (0.30)
Genital area	32985 (72.78)	1372 (50.97)
Other	477 (1.05)	
Total	45321	2692

CBA, Childbearing age; CSF, Cerebrospinal fluid; ORL,Otorhinolaryngological area; UTI, Urinary tract infection.

As for this cohort of pregnant women, GBS was associated in a case of choriamnionitis, preterm delivery and neonatal death. Other complications were 40 prelabor rupture of membranes, 44 urinary tract infections, 2 genital abscesses and 16 vaginal infections.

### Aggregated population isolation rate

The cumulative population exposed to infection by GBS during the study period (2009–2014) included 4,940,794 women born in Spain and 1,701,166 born abroad, with an average isolation rate of 5.19‰ (5.14–5.25‰). The isolation area is shown in Table 3.

**Figure 1:**
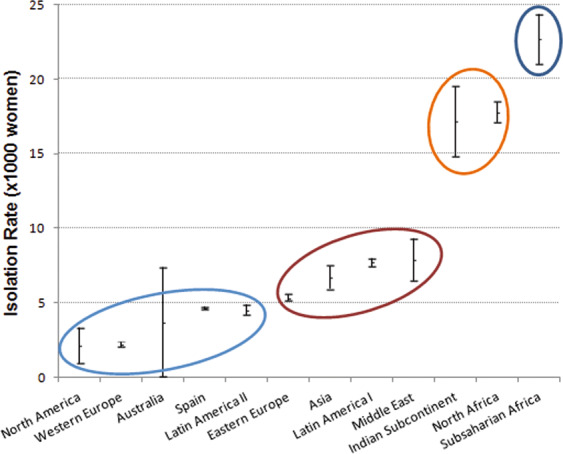
Isolation rates with 95% confidence intervals. Circles encompass the major groups derived from *post hoc* analysis.

**Figure 2:**
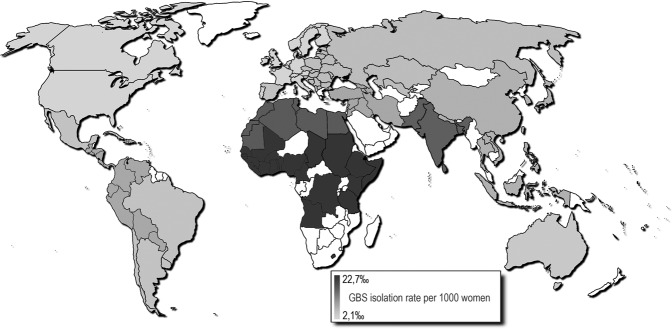
Global isolation rate map derived from aggregated data results.

The *post hoc* analysis showed that women from Sub-Saharan Africa had significantly higher GBS isolation rates compared to the rest of the groups: 22.7‰, whereas North America and Western Europe had the lowest: 2.1‰ and 2.2‰ respectively. The *post hoc* pairwise analysis found differences that allowed us to construct four major groups based on the significant differences between groups (Fig. 1). Global isolation rates are shown in Fig. 2.

### Co-variates

**Figure 3:**
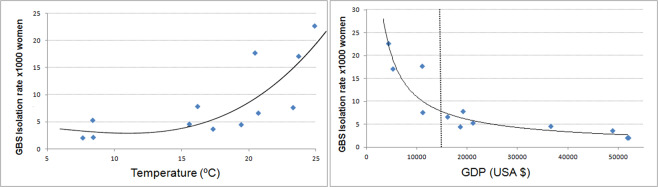
Temperature and GDP regression models.

Using aggregated population data, low GDP and high temperatures were associated with higher isolation rates, and fitted into non-linear regression models (Fig. 3). Temperature data by groups fitted into a cubic function with an R^2^ of 0.67 (F statistic = 8.34; p = 0.009); while GDP fitted into a potential function with an R^2^ of 0.88 (F statistic = 70.8; p < 0.00001). Using pregnant women data, a similar pattern was observed but R^2^ were smaller, 0.24 and 0.51 applying linear and potential models respectively.

**Figure 4:**
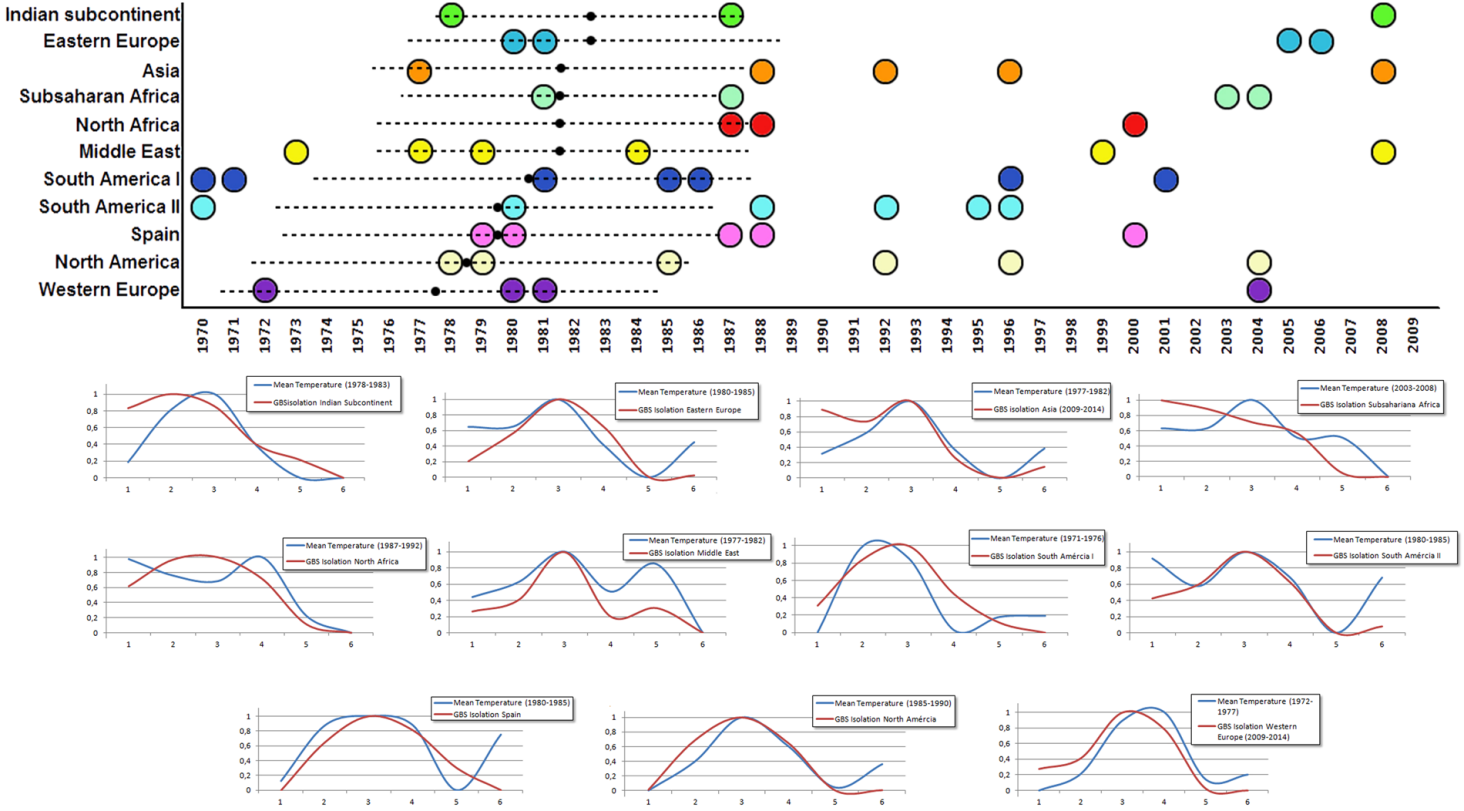
Correlation between temperature and GBS isolations trends. A coloured circle represents a match between temperature and GBS isolation trends, a black point represents the birth date of each population with a 95% confidence interval (dotted line). The best 6-year period matches are fully displayed for each geopolitical area.

Aggregated population GBS isolation trends and temperature trends presented several matches for each geopolitical area from 1970 to 2014. Figure 4 displays the years where both trends were alike, each coloured dot representing a 6-year period match. The birth date of each population with a 95% confidence interval is represented by a black point and a dotted line, and it was calculated by subtracting the mean age of each group to the middle year of the study period. The best fits between temperature and GBS isolation trends for each geopolitical area except Australia –due to the scarcity of data– are also displayed. The highest concentration of matches was found between the late 70 s and early 80 s, coinciding with the age around the birth date of the studied population. Developed countries presented better fits.

Higher income countries presented higher ages (p < 0.001), but plausible regression models to consider age as an explanatory co-variate yielded poor R^2^ values below 0.44 and 0.36 for aggregated population and pregnant women respectively.

## Discussion

The main finding of our study is that there are evident differences in GBS isolation among women of childbearing age depending on their country of origin; Africa and the Indian subcontinent presented the highest rates, while Western Europe and North America had the lowest. And more relevant, these differences have a mirror image in pregnant women. Also, this variability portrays a geographical pattern that seems influenced by temperature and GDP.

Variations in GBS colonization rates in population from different countries have been previously described; however, these studies offer different results and the total number of subjects studied is smaller and not comparable to ours. Three of these studies grouped women by geographical world regions but their results are not alike. From them, we can derive that Africa has a high GBS colonization rate, although their results vary and describe different geographical areas: i.e., 29% in Africa in Valkenburg-van den Berg *et al*., 21.8% in North Africa in Ramos *et al*., and 19% in Sub-Saharian Africa in Stoll *et al*.^24–26^. A fourth paper also describes different rates among Israeli and immigrant women, but their results are very local and the number of women included in the study is 681^13^. A systematic review focused on GBS maternal carriage in European countries found lower colonization rates in Western Europe compared to Eastern Europe^27^. But this study also compared two more groups, Scandinavia and Southern Europe, yielding results that do not match ours, especially the high colonization rate they report for Scandinavia (data not shown). Two more studies found differences in the USA in women and newborns from the same country according to their ethnic group^28,29^. Especially interesting are the results of Zaleznik *et al*., as they found that being black or Hispanic was a risk factor for developing neonatal disease^29^.

Regarding pregnant women from Hospital La Fe, our data correlate with that of the aggregated population. Actually, results for women from Africa are worrisome given that colonization rates are as high as 31% for Sub-Saharan countries and 21% for Northern Africa, which would explain Berardi *et al*.’s finding that African ethnicity was a risk factor for neonatal intrapartum GBS-transmission^5^. This means that, as long as our results are applicable to the original countries of the women included in the study, about one quarter of infants born in Africa would be at risk of GBS perinatal infection. Knowing that screening for GBS and intrapartum antibiotic prophylaxis are expensive and difficult to implement in lower-middle-income countries, such high colonization rate countries would benefit from vaccination of pregnant women as an alternative solution for both early and late onset GBS invasive infection, given that the vaccine included the appropriate serotypes^6,12,30–34^.

Age showed a slight effect on isolation rates, but contrary to mean temperature and GDP, it did not seem to play a major role in GBS colonization.

Humans, as homoeothermic beings, tend to keep a constant body temperature, but increased environmental temperature and atmospheric humidity have been shown to increase GBS carriage^35^. Features related to temperature such as clothing, skin and mucous moisture or even microbiota interactions may be crucial and modulate GBS colonization such as the inhibition of GBS by different strains of *Lactobacillus acidophilus* and *L*. *brevis*^36^. Interestingly, we found that the period of time around the birth of the studied population was the period were more correlations between isolation and temperature trends were found, suggesting that colonization during early life is paramount for future GBS carriage. Moreover, better fits were found for developed countries, maybe pointing out that, in political and economical stable areas, temperature becomes the most important driver for GBS colonization. Nevertheless, our data studied a 6-year period and, although tempting, it is too short a lapse of time to draw any conclusion. Temperature data over almost 50 years presented a nonlinear raising trend in all geopolitical areas; therefore, longer study periods are needed to observe such a trend in colonization rates. But if our data is right, poor countries, especially sub-Saharan countries could face a worrisome threat in years to come, and the development of effective vaccines could be the answer.

Interestingly, GDP appears to be paramount in our results since it was able to explain about 90% of the variation found in our data. Furthermore, we were expecting a linear co-relation between GDP and GBS colonization; but instead, we found an exponential one. This suggests that colonization levels can be reduced to a great extent if GDP reaches a certain point that, according to our data, may be at about 15000 USD (vertical, dotted line in Fig. 3B). Needless to say that an increase in GDP is linked to a safer and more regular food supply, better-quality sanitation and enhanced healthcare systems. Moreover, given the significance of GDP, it is likely to find different colonization rates among social classes with different incomes within a country. Thus, we wonder if the different colonization rates found by Regan *et al*. and Zaleznik *et al*.^28,29^ among white, black and Hispanic women in multicentre studies in the USA were due to the fact that white women tend to be in the upper and middle classes, whereas black and Hispanic women tend to be in the middle and lower classes^37,38^.

One weakness of our work is that we grouped countries to gain statistical power; thus, results are applicable for the whole group but should not be used to predict colonization rates for individual countries. Nevertheless, countries within each group for which there was enough data, followed suit the trends of the group they were included in. In addition, large countries such as the USA, Canada, Russia or China most certainly experience internal geographical variation. Another weakness is that people around the world have different reasons to migrate; hence, people from developing countries come mostly from the working class, whereas people from developed countries are most likely middle and upper class. Consequently, immigrants may arrive with different colonization rates according to their social class and their income as we have previously proposed.

In contrast, the main strength of our study is the large amount of data we could work with. Moreover, data from pregnant women fitted our results for the aggregated population, showing minor divergences in the order of colonization rates mainly in the middle rate groups, where differences are subtle and data were less abundant.

Our study has found important differences in GBS isolation rates according to the country of origin, as well as two likely explanatory reasons: GDP and mean temperature of the country of birth, given that GBS colonization is remarkably homogeneous and stable through time^13,14^. On the one hand, we offer current information on where international collaboration is needed and what should be done in order to diminish GBS-related infant morbidity and mortality; i.e., incrementing GDP in low-income countries and developing a future vaccine against GBS. On the other, we point out scenarios such as population displacements due to famine, natural disasters or war, where GBS colonization rates could increase rapidly and become an additional problem, especially for infants.

Another strength of our paper is that it shows new lines of investigation, i.e., how microbiota and GBS colonization may vary after moving to a different country. This task was beyond the scope of our paper due chiefly to the large amount of people included in this study. Moreover, our research overcomes possible doubts about GBS isolation due to intermittent vaginal colonization as data from pregnant women is consistent with the isolation rates found in the aggregated population.

Finally, our paper presents remarkable information for a global world, which should help to better treat pregnant women and prevent infant mortality. But this is just the beginning, our results should be compared to local data from countries to picture the actual situation accurately and help scientists to develop new lines of research.

## Conclusion

Our paper includes a large population dataset of childbearing age women from almost every major area of the globe which represents a mini picture of the world. Therein, we have found regional trends in GBS colonization that are linked to income and, to some extent, to climate. Finally, this information could help identify populations at higher risk of developing perinatal or post-partum GBS infection in their new countries, and to some extent, points out possible fields of action to avoid such events.
